# Advances in Our Understanding of “Resistance” to Antiplatelet Agents for Prevention of Ischemic Stroke

**DOI:** 10.1155/2013/727842

**Published:** 2013-07-14

**Authors:** Philip B. Gorelick, Muhammad U. Farooq

**Affiliations:** ^1^Hauenstein Neuroscience Center, 220 Cherry Street SE, Grand Rapids, MI 49503, USA; ^2^Department of Translational Science and Molecular Medicine, Michigan State University College of Human Medicine, 333 Bostwick Avenue NE, Grand Rapids, MI 49503, USA

## Abstract

We review the role of aspirin and clopidogrel for prevention of ischemic stroke and explore the concept of antiplatelet therapy resistance both from a laboratory and clinical perspective and genetic polymorphisms that might influence platelet reactivity with clopidogrel administration. Debates have raged over the years about the application of platelet function tests in clinical practice. We conclude that platelet function testing is not indicated in routine clinical practice. This recommendation is supported by clinical guideline statements, a lack of a global platelet function measure, and limitations of current platelet function test methods as applied in practice. We discuss a recently hypothesized hierarchy of patient characteristics in relation to which patients are most likely to benefit from platelet function studies based on acuity (i.e., risk) of cardiovascular disease. A focus of antiplatelet therapy administration should include emphasis on compliance/adherence and in the example of aspirin, use of well-absorbed forms of aspirin and avoidance of drugs that may interact with aspirin to inhibit its mechanism of action (e.g., certain nonsteroidal anti-inflammatory drugs).

## 1. Introduction

In the 1700s, Edward Stone, a clergyman, pulverized the bark of the willow and created a tea that reduced fever [1]. Some 70 years later, salicin or salicylic acid, was identified as the active ingredient of the therapeutic brew. The active substance, however, was known to be bitter and irritating to the stomach. In the mid to late 1800s, a less toxic form of the agent was synthesized, and in 1898 Felix Hoffmann was credited for synthesizing acetylsalicylic acid, later named “aspirin” whereby the “a” denoted acetyl and “spirin,” *Spiraea ulmaria*, the source of salicylic acid [1, 2]. In the era of modern cardiovascular prevention, Lawrence Craven reported that aspirin might prevent coronary artery thrombosis and ischemic stroke, but also might lead to hemorrhagic complications [3, 4]. Craven's insights were the forerunners to large scale, randomized controlled trials (RCTs) of aspirin in the prevention of cerebral ischemia and cardiovascular disease. It was not until the 1970s that John Vane elucidated the mechanism of aspirin—suppression of biosynthesis of prostaglandins [5]. Since that time we have witnessed a substantial growth of RCTs featuring aspirin and newer antiplatelet therapies for stroke and cardiovascular disease prevention. 

Aspirin has withstood the test of RCTs over time as a cost-effective approach to stroke and cardiovascular disease prevention [6]. The effectiveness of aspirin and other antiplatelet agents, however, has been subject to criticism related to possible “resistance” or biologic variability. In this discussion we provide a brief update of the latter topic in relation to aspirin and clopidogrel. We have chosen to review these two agents based on frequency of use in practice and availability of study data. Our review is not a formal meta- and systematic analysis but rather includes scientific data known to the authors in their personal study files. 

## 2. Aspirin: Guidelines for Use in Stroke Prevention

We begin our discussion with a brief review of United States (US) guidelines to show the position of aspirin in stroke prevention. The American Heart Association (AHA) guidelines recommend an initial dose of aspirin 325 mg within 24 to 48 hours after ischemic stroke onset for treatment of most patients (Class I; Level of Evidence [LOE] A), and after 24 hours for those who have received intravenous fibrinolysis [7]. Similarly, the American College of Chest Physicians (ACCP) guidelines recommend early (48 hour) aspirin treatment at an initial dose of 160 to 325 mg (Grade 1A) for persons with ischemic stroke or TIA [8]. In addition, in both sets of guidelines aspirin mono-therapy at a dose of 75 to 100 mg/day (Grade 1A) [8] or 50 to 325 mg/day (Class I; LOE A) [9] is one of the recommended acceptable initial antiplatelet agents to reduce the risk of recurrent stroke and other cardiovascular events for patients with non-cardio-embolic ischemic stroke. 

The recommendation for primary prevention of cardiovascular disease slightly differs between US ACCP and AHA guidelines. Specifically, ACCP recommends low-dose aspirin, 75 to 100 mg/day for persons 50 years of age and older (Grade 2B) [8], whereas AHA guidelines recommend aspirin prophylaxis for persons at sufficiently high risk when the benefits outweigh the risks of treatment (e.g., 10-year risk of cardiovascular events of 6 to 10%; Class I; LOE A); aspirin in a dose of 81 mg/day or 100 mg every other day may be useful for first stroke prevention among women who have a sufficiently high enough benefit to risk equation (Class IIa; LOE B); and aspirin is not indicated for those at low risk (Class III; LOE A)and for preventing a first stroke in those with diabetes or diabetes plus asymptomatic peripheral artery disease when there is no established cardiovascular disease (Class III; LOE B) [10]. 

Furthermore, the Antithrombotic Trialists' (ATT) Collaboration assessed the benefits and risks of aspirin in primary prevention from 6 trials [11]. In one phase of the analysis they compared long-term aspirin versus control therapy in 6 primary prevention trials of 95,000 persons at low risk over 660,000 person-years that included 3554 vascular events. Overall, aspirin was associated with a 12% proportional reduction in serious vascular events (*P* = 0.0001) mainly due to a reduction by about 20% of non-fatal myocardial infarction though the net effect on stroke was not significant (*P* = 0.4) (hemorrhagic stroke 0.04% versus 0.03%, *P* = 0.05 and other stroke 0.16% versus 0.18%, *P* = 0.08) [11]. Major gastrointestinal and extracranial bleeds, however, were increased (0.10% versus 0.07%, *P* < 0.0001). In addition and overall, in the primary prevention trials, the proportional reduction in serious vascular events did not depend substantially on age, sex, smoking history, blood pressure, total cholesterol, body mass index, history of diabetes mellitus or risk of coronary heart disease. 

In a more recent meta-analysis of 9 RCTs in the area of primary prevention that included 102,621 patients followed over 6 years, aspirin was estimated to significantly reduce non-fatal myocardial infarctions by about 20% and total cardiovascular events by 10% without a substantial reduction in death or cancer [12]. Furthermore, the risk of non-trivial bleeds was 31% higher among those who received aspirin therapy, and was believed to offset the benefits. The authors concluded that aspirin in primary prevention was not indicated based on a number-needed-to-harm of 73 for non-trivial bleeding events that dwarfed any benefits. Currently, there is interest in aspirin as a therapy to prevent deaths in cancer and distant metastases [13]. These observations are important ones, and genetic and molecular mechanisms of these possible effects are being elucidated. It has been hypothesized that aspirin administration for up to 5–10 years may be required before a beneficial effect on cancer risk reduction is observed [14]. 

## 3. Clopidogrel: Guidelines for Use in Stroke Prevention

In both the ACCP and AHA guidelines for recurrent stroke prevention, clopidogrel 75 mg/day is considered an acceptable initial option for non-cardioembolic recurrent ischemic stroke prevention (Grade 1A; Class I, LOE A, resp.) [8, 9]. Clopidogrel is not considered a first-line agent for first stroke prevention [10]. 

## 4. Metabolism, Resistance, and Laboratory Testing for Resistance to Aspirin and Clopidogrel

### 4.1. Metabolism of Aspirin

 Aspirin (acetylsalicylic acid) is quickly absorbed from the stomach and upper small intestine by passive diffusion and reaches peak plasma levels in about 30–40 minutes after administration of the immediate-release oral formulation [15]. Enteric-coated preparations, however, may take up to 3-4 hours to achieve peak plasma levels. The oral bioavailability of aspirin is approximately 40 to 50% over a range of doses, whereas for enteric-coated and sustained-release preparations, it is substantially lower. The portal circulation is the first point of contact of aspirin with platelets, and the half-life of aspirin is 15 to 20 minutes [15]. Despite a short half-life, there is permanent inactivation of the platelet for its entire life. At the cellular level, aspirin inactivates the cyclooxygenase (COX) activity of prostaglandin H (PGH) synthase 1 (COX-1) and synthase 2 (COX-2). Thus, the conversion of arachidonic acid to PGH2 is affected and several downstream bioactive prostanoids such as thromboxane A2 (TXA2), a vasoconstrictor, inducer of vascular smooth muscle, a pro-atherogenic factor, and platelet aggregant, and prostacyclin (PGI2) which has essentially opposite effects to TXA2, are affected. Platelets produce TXA2 whereas the vascular endothelium produces PGI2. The balance between PGI2 and TXA2 is thought to be important. The molecular mechanism of inactivation of COX activity by aspirin is the blockade of a channel caused by acetylation of a serine residue, Ser529 on COX-1 and Ser516 on COX-2. Select details of platelet activation are listed in Table 1 [16]. 

### 4.2. Defining Resistance to Aspirin and Its Causes

 Aspirin resistance may be classified as a laboratory or clinical phenomenon [17]. Laboratory resistance may be defined as a failure to inhibit platelet TXA2 production or tests of platelet function (e.g., platelet aggregation) dependent on platelet TXA2 production. Clinical resistance may be defined as failure of aspirin to prevent clinical atherothrombotic events which also may be referred to as aspirin treatment failure [17]. Traditionally in biologic systems, drug resistance is defined as being caused by microbes, viruses or cancer cells that change to reduce or eliminate a drug's effectiveness or genetic changes alter drug targets such as enzymes or transmembrane proteins that lead to reduced or no drug activity [18]. Thus, aspirin “resistance” differs from the traditional definition of resistance in that the change is not in the drug target per se as in the traditional use of the term. Furthermore, the effects may fluctuate and are at least partially reversible by changing the dose of aspirin [18]. 

Hankey and Eikelboom discuss possible causes for aspirin failure and include the following categories: (1) Reduced bioavailability (e.g., poor compliance or drug not prescribed, reduced absorption or metabolism); (2) Altered binding to COX-1 (e.g., ibuprofen administration); (3) Other sources of TXA2 production (sources from monocytes, macrophages and endothelial cells); (4) Alternative pathways of platelet activation (e.g., increased sensitivity of platelets to collagen and ADP); (5) Increased platelet turnover (increased platelet production by bone marrow in response to coronary artery bypass surgery); (6) Genetic polymorphisms (e.g., polymorphisms of COX-1, COX-2); (7) Loss of antiplatelet effect with long-term administration of aspirin (tachyphylaxis); and (8) Non-atherothrombotic causes of cardiovascular events not expected to respond to antiplatelet agents (e.g., vasculitis) [17]. 

### 4.3. Diagnosing Laboratory Resistance to Aspirin

 The frequency of laboratory resistance to aspirin has been estimated to be up to 61% [17]. The estimates vary substantially based on disparate study populations, overall methods, and specific tests of platelet function. Importantly, as many as 40% of patients with cardiovascular disease may not be compliant with aspirin therapy. 

Platelet function can be assessed by point-of-care and other laboratory tests. Most of the tests are *ex vivo* ones [18].  Table 2 provides a listing of the tests and a brief commentary about them. Thus far, point-of-care or other platelet function tests or genetic tests have not been mandated for use in practice according to guideline statements [18, 19]. Such testing has been employed in practice and research, but has not been considered a mandatory part of practice. In the case of clopidogrel, for example, one guidance statement concluded that genetic tests to detect poor metabolizers at moderate or high risk for poor outcomes may be considered [19]. As we will discuss below, these tests may have more value in certain clinical circumstances. 

### 4.4. Metabolism of Clopidogrel

 Clopidogrel is a prodrug that must be converted to its active form in the liver [20, 21]. Once ingested clopidogrel is absorbed in the intestine whereby absorption may be limited by P-glycoproteins encoded by the ABCB1 gene. Most of the drug (about 85%) is metabolized by esterases into an inactive form, whereas the remainder is converted from the prodrug to the active state at the cytochrome P450 (CYP) site by active isoforms. Whereas aspirin inhibits COX, clopidogrel irreversibly inhibits the adenosine diphosphate (ADP) receptor coded by the P2RY12 gene responsible for inactivating the fibrinogen receptor, glycoprotein IIb/IIIa, responsible for platelet aggregation. CYP-dependent oxidative steps are critical for the conversion of the prodrug to its active form, and carriage of certain CYP2C19 and CYP3A4 alleles, for example, may be associated with response to clopidogrel as oxidative-dependent metabolism of clopidogrel occurs. The CYP2C19 polymorphisms include a *1 normal function isoform and *2 and *3 loss of function alleles. Poor metabolizers may be defined as those having two loss of function alleles and intermediate metabolizers as those with one loss of function allele. In addition, a gain-in-function allele, CYP2C19*17 exists and serves as a hyper- or ultra-rapid metabolic pathway for the conversion of clopidogrel to its active form. Certain drugs such as proton pump inhibitors (PPIs) may use the same pathway of liver metabolism as clopidogrel, and thus, may be associated with diminished clopidogrel response [21]. 

### 4.5. Diagnosing Laboratory Resistance to Clopidogrel

 In the example of clopidogrel, possible laboratory resistance may be defined by platelet function tests or genetic tests.  Table 2 lists and reviews platelet function tests that may be used to define clopidogrel resistance and the limitations of these tests [18, 21]. Such tests as VerifyNow, Thromboelastography, PFA-P2Y, and the degree of phosphorylation of VASP may be employed. 

Loss of function alleles for the conversion of clopidogrel from the prodrug to its active form have been associated with diminished platelet inhibition and poorer outcomes in relation to occurrence of major events in persons with acute coronary syndromes or percutaneous coronary intervention [21–24]. The clinical circumstances are complex as multiple factors may be involved in the attribution of clopidogrel metabolism. For example, in high-risk coronary patients, loss-of-function alleles may occur in up to 20%, and it is estimated that at least one copy of the reduced function CYP2C19*2 allele occurs in 50% of Chinese, 34% of African Americans, 25% of Whites, and 19% of Mexican Americans [19, 21]. Furthermore, there may be polymorphisms of the P2Y12 receptor such that there is decreased affinity to clopidogrel, and drugs such as rifampin may lead to increased activation of the CYP3A4 site, and other drugs such as PPIs may compete with clopidogrel at the CYP metabolic activation site [21]. One specific PPI, omeprazole, has been cited by the US FDA in a “Black Box Warning” statement as a concern for administration with clopidogrel, though the topic remains controversial [25]. 

### 4.6. Summary Thoughts about Laboratory Resistance to Aspirin and Clopidogrel

 Overall, and as summarized by a number of authors in relation to aspirin and other antiplatelet agent laboratory testing to detect resistance, there may be considerable differences between point-of-care and other platelet function test results. Therefore, these tests require additional prospective study in large trials and observational studies before they will be ready for routine use in clinical practice [18, 21, 26–28]. After reviewing the arguments for and against platelet function monitoring tests, we are most impressed by the lack of a suitable global platelet test measure and clinical supporting evidence that the results of such testing will clearly make a difference in stroke prevention management [29–34]. 

### 4.7. New Clinical Information

Adjustment of aspirin dose to higher levels to provide more effective prevention of cerebrovascular disease has long been debated [35], however, over time most agree that lower doses of aspirin (e.g., 50–325 mg/day) provide similar point estimates of stroke or composite stroke, myocardial infarction or vascular death reduction as higher doses, but lower dose aspirin is safer [2]. Opportunity to get the most benefit out of aspirin and other antiplatelet agents may be as simple as encouraging compliance or adherence, and in the case of aspirin, avoiding use of certain concomitant drugs, when possible, such as ibuprofen which may competitively inhibit low-dose aspirin from leading to an irreversible inhibition of platelet function [36]. Or, by avoiding enteric-coated aspirin which may lead to delayed or reduced aspirin absorption [37]. 

Intensification of platelet inhibition and reduction of residual platelet activity in patients with acute coronary syndrome by treatment with clopidogrel or newer antiplatelet gents such as prasugrel or ticagrelor has been a recent focus of interest [38–40]. Interestingly, recent studies have shown no significant improvements in clinical outcomes with platelet function monitoring and treatment adjustment when there is coronary artery stenting [41]. Furthermore, among patients with acute coronary syndrome without ST-segment elevation in a platelet sub study, there was no significant association between platelet reactivity and major ischemic outcomes [42]. These studies cast further doubt on the clinical usefulness of platelet function monitoring in practice. Similar skepticism has been leveled against the CYP2C19 genotype and occurrence of cardiovascular events when clopidogrel is used [43, 44]. 

Currently, there is new evidence to suggest that the association between P2Y12-mediated platelet reactivity and clinical outcomes may depend on the clinical context within which platelet function is measured [45]. For example, there may be a hierarchy by disease acuity whereby platelet function becomes more meaningful. Specifically and in relation to coronary artery disease, patients with coronary syndromes requiring acute percutaneous coronary intervention (PCI) may be most affected by adverse outcomes (e.g., stent thrombosis) when there is on-treatment platelet reactivity, whereas the risk is less for those undergoing PCI for stable coronary disease, and is not strongly associated with outcomes in medically treated patients [42, 45]. Finally, the evidence base has expanded in relation to clopidogrel and cigarette smoking status to suggest a reduced or complete lack of clinical benefit in association with clopidogrel use in nonsmokers [46]. The explanation for this phenomenon is thought not to be an enhanced prothrombotic state in smokers but rather by an induction of activity of the CYP1A2 isoenzyme in smokers that leads to metabolic activation of clopidogrel. These two observations [45, 46] may help us to focus our study for meaningful clinical use of platelet function tests in stroke and cardiovascular disease practice. 

## 5. Conclusion

“Resistance” to antiplatelet therapy has been defined as a laboratory or clinical phenomenon. Laboratory resistance may include failure to inhibit platelets based on platelet function tests or laboratory evidence of failure to inhibit the metabolic pathway that should be inhibited by a given drug. Clinical resistance may be defined as failure to prevent meaningful clinical atherothrombotic events which also may be referred to as treatment failure. Platelet function testing remains a clinical research tool. At this time, it is not recommended for routine clinical use as there is no global platelet function measure, and there are significant limitations of testing (see Table 2). As the science of platelet function testing advances, we are beginning to target groups of patients that might be more likely to benefit from such testing [45]. These patients preferentially may be those with acute cardiovascular disease, especially those undergoing acute revascularization interventions. 

Conversion of c lopidogrel from its prodrug state to its active metabolite may be affected by a number of factors of which cigarette smoking may be one of them [46, 47]. Specifically, there has been a body of emerging evidence that shows a concordance between cigarette smoking, greater pharmacodynamic efficacy, and clinical response to clopidogrel therapy [47, 48]. Encouragement of cigarette smoking is not a public health option, however, newer more potent antiplatelet agents such as prasugrel and ticagrelor that are associated with better clinical outcomes than clopidogrel in high-risk coronary artery disease patients may be administered, if bleeding risk is permissive [48]. In relation to stroke, prasugrel and ticagrelor are not labeled for use by the US FDA and may be associated with brain bleeding risk. As for aspirin, adherence remains a challenge (47% or less in the US) and is an important point of informed discussion between the patient and healthcare provider about competing risks of bleeding and reduction of stroke and cardiovascular events [49]. Increasing the aspirin dose [50] or changing from one antiplatelet agent to another (e.g., aspirin to clopidogrel or clopidogrel to aspirin) has not been definitively shown to prevent subsequent recurrent stroke [9]. Therefore, in relation to aspirin, a focus on adherence and use of drugs that may alter aspirin absorption or effect makes sense in practice. 

## Figures and Tables

**Table 1:** Select steps in platelet activation [16].

	(1) Receptor complexes tether platelets to sites of vascular injury: glycoprotein Ib/V/IX and platelet surface collagen receptors glycoprotein VI and Ia
	(2) Mediators of adhesion phase, and amplification and sustenance of platelet response: adenosine diphosphate (ADP), thrombin, epinephrine, and TXA2
	(3) Final activation pathway by involvement of agonists: activation of platelet integrin glycoprotein IIb/IIIa receptor for adhesion and aggregation

TXA2: thromboxane A2.

**Table 2:** Platelet function tests and commentary [18, 21].

(1) Thromboxane A2 synthesis	Measurement of metabolites such as serum thromboxane B2 or urinary 11-dehydro-thromboxane B2, direct metabolites of COX-1, a specific mechanistic target of aspirin may be made. These tests are limited by a nonlinear relationship between platelet COX-1 activity and thromboxane A2 activity, and extra-platelet sources of thromboxane A2 synthesis. Furthermore, urinary excretion of 11-dehydro-thromboxane B2 must be normalized according to urinary function (e.g., creatinine concentration).

(2) Aspirin response according to thromboxane dependent assays light transmittance aggregometry (LTA), impedance aggregometry (IA), platelet function analyser (PFA-100), and VerifyNow	LTA measures light transmission through a platelet suspension exposed to a platelet agonist such as ADP, but the agonists may activate pathways less dependent on COX-1. IA measures electrical impedance after exposure to whole blood suspension by a platelet agonist. There may be poor reproducibility, variation of response by age, race, sex, hematocrit, and concentration of the agonist. Like IA, LTA may be associated with poor reproducibility. PFA-100, an *in vitro* recorder, includes a membrane with an aperture coated with collagen plus an agonist (e.g., epinephrine, ADP). As platelets form aggregates, the aperture occludes, and flow factors may affect test results (e.g., nonsteroidal anti-inflammatory drugs, clopidogrel, GP IIb/IIIa expression on the platelet surface, von Willebrand factor, platelet count, hematocrit, and diurnal variation (lower closing times in the morning)). VerifyNow measures platelet function by light transmission through a suspension of lyophilized fibrinogen-coated beads and an agonist such as arachidonic acid. *Clopidogrel Response*. Platelet function measured by LTA using the agonist, ADP, before and after treatment, is the main standard test to assess clopidogrel. Point-of-care assays such as VerifyNow, Thromboelastography (discussed below), and PFA-P2Y may be employed. These tests all have limitations that are discussed elsewhere (see [21]).

(3) Thromboelastography	Measures the contribution of ADP-induced aggregation to tensile strength of platelet-fibrin clot and requires further validation studies as does the PFA P2Y test that measures clopidogrel response.

(4) Degree of phosphorylation of VASP	Clopidogrel irreversibly blocks the ADP receptor P2Y12 and activates a cAMP-dependent protein kinase a that inhibits VASP, vasodilator-stimulated phosphorylation. VASP is an inducer of platelet aggregation via GP IIb/IIIa. The degree of phosphorylation of VASP to an antiplatelet agent may be determined by flow cytometry, but there may be limited sensitivity.
